# Identification of a Four-Gene Signature Associated with the Prognosis Prediction of Lung Adenocarcinoma Based on Integrated Bioinformatics Analysis

**DOI:** 10.3390/genes13020238

**Published:** 2022-01-27

**Authors:** Yuan Wu, Lingge Yang, Long Zhang, Xinjie Zheng, Huan Xu, Kai Wang, Xianwu Weng

**Affiliations:** 1 Department of Respiratory Medicine, The Fourth Affiliated Hospital, College of Medicine, Zhejiang University, Yiwu 322000, China; yuanwu@zju.edu.cn (Y.W.); lingeryang93@zju.edu.cn (L.Y.); zzzhanglong@zju.edu.cn (L.Z.); zhengxinjie@zju.edu.cn (X.Z.); m15268336009@163.com (H.X.); 2 Department of Cardiothoracic Surgery, The Fourth Affiliated Hospital, College of Medicine, Zhejiang University, Yiwu 322000, China

**Keywords:** lung adenocarcinoma (LUAD), weighted gene co-expression network analysis (WGCNA), least absolute shrinkage and selectionator operator (LASSO), gene signature, prognosis prediction

## Abstract

Lung adenocarcinoma (LUAD) is often diagnosed at an advanced stage, so it is necessary to identify potential biomarkers for the early diagnosis and prognosis of LUAD. In our study, a gene co-expression network was constructed using weighted gene co-expression network analysis (WGCNA) in order to obtain the key modules and genes correlated with LUAD prognosis. Four hub genes (HLF, CHRDL1, SELENBP1, and TMEM163) were screened out using least absolute shrinkage and selection operator (LASSO)–Cox regression analysis; then, a prognostic model was established for predicting overall survival (OS) based on these four hub genes..Furthermore, the prognostic values of this four-gene signature were verified in four validation sets (GSE26939, GSE31210, GSE72094, and TCGA-LUAD) as well as in the GEPIA database. To assess the prognostic values of hub genes, receiver operating characteristic (ROC) curves were constructed and a nomogram was created. We found that a higher expression of four hub genes was associated with a lower risk of patient death. In a training set, it was demonstrated that this four-gene signature was a better prognostic factor than clinical factors such as age and stage of disease. Moreover, our results revealed that these four genes were suppressor factors of LUAD and that their high expression was associated with a lower risk of death. In summary, we demonstrated that this four-gene signature could be a potential prognostic factor for LUAD patients. These findings provide a theoretical basis for exploring potential biomarkers for LUAD prognosis prediction in the future.

## 1. Introduction

Lung cancer is the third most common cancer worldwide and has the highest mortality rate. Nearly 1.8 million new cases and 1.6 million lung cancer-related deaths are reported every year [1]. As the most prevalent subtype of lung cancer, lung adenocarcinoma (LUAD) occurs more frequently in smokers. However, in recent years, non-smoker morbidity from LUAD has markedly increased [2]. Due to the lack of efficient diagnostic methods, LUAD is often diagnosed at an advanced stage. Despite the rapid development of gene targeted therapy and immunotherapy leading to a significant improvement in patient survival rates and quality of life, the 5-year mortality rate of LUAD is still very high, ranging from 51% to 99% depending on the disease stage [3]. Therefore, it is necessary to understand the molecular mechanism of LUAD development and to identify novel potential biomarkers for the early diagnosis and prognosis of LUAD.

Over the past two decades, the oncogenesis mechanisms of lung cancer have come to be largely understood. It is now widely known that lung cancer is a molecularly heterogeneous disease that features alterations of oncogenes and tumor suppressor genes [4]. A growing number of oncogenic alterations have been identified in non-small cell lung cancer (NSCLC), such as EGFR, ALK, and ROS1. These oncogenic alterations promote the fast development of small molecule-targeting inhibitors for lung cancer therapy [5,6,7]. Moreover, previous studies have revealed that these alterations play essential roles in various pathological processes of LUAD, including tumor progression and metastasis [8,9]. Recently, extensive investigations of the tumor immune microenvironment have facilitated the application of immunotherapy in clinical settings and have dramatically changed the landscape of lung cancer treatment. Programmed death-ligand 1 (PD-1/PD-L1) inhibitors are two main immune checkpoint inhibitors (ICIs) that are applied in the clinic. PD-1 signaling, mainly driven by adaptive PD-L1 expression in tumors, inactivates T cells that identify tumor-specific antigens and promotes tumor progression and metastasis [10,11]. PD-L1 and the tumor mutation burden (TMB) are two independent predictors of responses to immunotherapy [12,13]. Nevertheless, the precise molecular mechanisms of lung cancer are far from being elucidated and more potential prognostic biomarkers are required. In recent years, several studies have identified candidate genes for potential therapeutic targets and diagnostic/prognostic biomarkers of lung cancer through bioinformatics analysis. For example, it was reported that seven genes were analyzed as potential diagnostic biomarkers for NSCLC based on the Gene Expression Omnibus (GEO) and The Cancer Genome Atlas (TCGA) databases [14]. Moreover, the dysregulation of non-coding RNAs, including long non-coding RNAs (lncRNAs) and miRNAs identified by bioinformatics analysis, was found to be associated with carcinogenesis [15]. Though much progress has been made in the treatment of LUAD, it is still necessary to identify putative biomarkers for the precise diagnosis and prognosis valuation of LUAD patients.

In this study, gene datasets including information on sample sizes and prognostic information for LUAD were downloaded from the GEO and TCGA databases. We aimed to screen out key modules and genes correlated with LUAD prognosis through weighted gene co-expression network analysis (WGCNA) and least absolute shrinkage and selection operator (LASSO) regression analysis. Four hub genes that significantly correlated with overall survival were selected and a four-gene signature was created for LUAD prognosis. The prognostic value of this four-gene signature was verified in various validation sets. Additionally, this signature showed a desirable sensitivity and specificity for predicting the overall survival (OS), as well as the disease-free survival (DFS), of LUAD patients.

## 2. Materials and Methods

### 2.1. Data-Collection and Pre-Processing

Series matrix files of GSE30219 [16], GSE37745 [17], and GSE50081 [18] were downloaded from GEO database (https://www.ncbi.nlm.nih.gov/geo/, accessed on 23 March 2021). These matrix files were all based on the platform of GPL570, so we merged these matrix files after a series of normalization, and the batch effects among these datasets were removed by using the “sva” package (versions 3.36.0) [19] of R 4.0.2. A training set was finally obtained for the following analysis. The datasets of GSE30219, GSE33745, and GSE50081 contained 85, 106, and 130 cancer tissues, respectively. A total of 321 LUAD cases were used for the following WGCNA and prognosis model building. In addition, we downloaded the series matrix files of GSE26939 [20], GSE31210 [21], and GSE72094 [22] from the GEO database, aiming to validate the prognosis model. The datasets of GSE26939S included 116 LUAD samples based on the platform of GPL9053, while the datasets of GSE31210 comprised 226 LUAD samples based on the platform of GPL570. Additionally, the datasets of GSE72904 included 442 LUAD samples based on the platform of GPL15048. Then, the RNA sequencing datasets of 500 LUAD cases with clinicopathological information were downloaded from the TCGA (https://cancergenome.nih.gov/, accessed on 25 April 2021) database. The FPKM (fragment per kilobase per million) level 3 data from the TCGA database were used in this study. All the information concerning the datasets used is listed in Table 1.

### 2.2. WGCNA to Screen Out a Key Module and Genes Related to Survival

The gene co-expression network was constructed using the “WGCNA” package (versions 1.70-3) [23]. According to the gene expression datasets of the training set, we utilized the goodSampleGenes method to remove non-expressed genes and to select expressed genes with a standard deviation of >1.2 for cluster analysis. Next, we started to build a scale-free co-expression network. Firstly, the Pearson’s correlation matrices and average linkage method were carried out for all pair-wise genes. Then, a weighted adjacency matrix was constructed using the power function, as follows:

A_mn_ = |C_mn_|^β

(C_mn_ = Pearson’s correlation between gene_m and gene_n; A_mn_ = adjacency between gene_m and gene_n).

β is a soft-thresholding parameter that can emphasize strong correlations between genes and penalize weak correlations. After choosing the power (β), the adjacency was transformed into a topological overlap matrix (TOM). TOM is able to measure the network connectivity of a certain gene, defined as the sum of its adjacency with all other genes, as a network gene ratio. Moreover, the corresponding dissimilarity (1-TOM) can be calculated. To classify genes with similar expression profiles into gene modules, average linkage hierarchical clustering was conducted according to the TOM-based dissimilarity measure. The minimum size (gene group) for the gene dendrogram was 30 and the minimum size for deepSplit was 2. DeepSplit is a parameter that can adjust the sensitivity of partition modules; the greater its value is, the more sensitive it is and the more modules are obtained. After we calculated the eigengenes of gene modules using the dynamic shear method, we used cluster analysis to merge close modules into new modules (height = 0.25). Then, correlations between the modules or genes in the modules and the phenotypes of the training sets were analyzed based on the eigenvectors of modules and gene expression of samples. Thereby, hub genes were selected for further prognosis model building (R > 0.7).

### 2.3. GO Enrichment and KEGG Pathway Analysis

Gene Ontology (GO) and Kyoto Encyclopedia of Genes (KEGG) pathway enrichment analyses of the key modules were performed based on the Database for Annotation, Visualization, and Integrated Discovery (https://david.ncifcrf.gov, DAVID, version 6.8, accessed on 3 May 2021) [24]. We used the “ggplot2” package (versions 3.3.5) [25] to plot the figures based on the GO enrichment analysis results (*p* value < 0.01) and the KEGG pathway analysis results (*p* value < 0.05).

### 2.4. Prognostic Genes Screening Associated with OS and DFS

After integrating the survival time, survival status, and gene expression data, a univariate survival analysis was performed using the “survival” package (versions 3.2-7) [26] for the OS and DFS of LUAD patients in the training sets. Then, we divided the LUAD patients into a high expression group and low expression group according to the median value of gene expression found. Selected genes were intersected with candidate genes in the key module, and the genes that would be used in eventual model were obtained. The results were plotted using online tool named Bioinformatics and Evolutionary Genomics (http://bioinformatics.psb.ugent.be/webtools/Venn/, accessed on 24 April 2021). *p* < 0.05 between the two groups was considered statistically significant.

### 2.5. Prognostic Gene Signature Construction and Validation

Gene expression matrix profiles and OS-related prognosis information were prepared in order to build the LASSO-Cox regression model using the “glmnet” package (versions 4.1-2) [27]. We set nfold = 15 and took λ as lambda.min in order to acquire an optimized model. The risk score for OS (RSO) was calculated based on the coefficients of genes, which affected the prognosis of LUAD. The results were equalized according to the following formula:

RSO = Coefficient 1 × gene 1 RNA expression + Coefficient gene 2 RNA expression + …+ Coefficient n × gene n RNA expression

Thus, the RSO values of each sample in the training set as well as in the validation sets were calculated. The gene expression heatmaps of RSO in each dataset were generated using the “ggplot2” package (versions 3.3.5) [25], and time-dependent (1-year, 3-year, and 5-year) receiver operating characteristic (ROC) and K-M curves were generated with the “timeROC” package (versions 0.4) [28]. To further verify the prognostic value of the built gene signature, we analyzed the four genes individually and together via the Survival Analysis module in the Gene Expression Profiling Interactive Analysis (http://gepia.cancer-pku.cn/, GEPIA, accessed on 28 April 2021) database [29]. Moreover, we analyzed the expression of the four genes in normal tissues and tumor tissues through the datasets.

### 2.6. Univariate and Multivariate Cox Regression Analysis

We used the “survival” package (versions 3.2-7) of R 4.0.2 [26] to integrate the survival time, survival status, RSO, and other common clinical characteristics (age, sex, clinical stages) of all datasets. A univariate analysis was performed using the Cox method. Variables with a *p* < 0.05 were included in a multivariate Cox regression analysis.

Forestplot, Nomogram, and calibrated curves were created using the “forestplot” package (versions 1.10) [30] and “rms” package (versions 6.2-0) [31]. The forestplot was obtained based on the clinical information of patients, HR, and 95%CI in univariate and multivariate regression analyses. The nomogram was composed of previously screened independent prognostic factors and internally validated by bootstraps with 1000 resamples. Every factor was assigned a weight based on its effect on the prognosis. Thus, the corresponding score was acquired, allowing us to predict the 1-, 3-, and 5-year survival probability of LUAD patients according to the weight of each factor. Generally, a higher score represented a worse prognosis. The calibration curve was obtained based on the real survival of patients and was used to predict the probability of survival in a nomogram. When the predicted probability is close to the real survival status, the calibration curve is more likely to be diagonal. Therefore, if the broken line fluctuates near the diagonal, this indicates that the fit of the prediction model is good.

### 2.7. Gene Set Enrichment Analysis (GSEA)

The basic idea of Gene Set Enrichment Analysis (https://www.gsea-msigdb.org/gsea, GSEA, accessed on 19 May 2021) [32] is to use a predefined set of genes. The training set was divided into a high-risk group and low-risk group depending on the cutoff value of the RSO. Then, we conducted hallmark and KEGG pathway analyses using GSEA to further analyze the possible pathways involved between these two risk groups. Subsets of c2.cp.kegg.v7.4.symbols.gmt and h.all.v7.4.symbols.gmt were downloaded to help us evaluate the pathways and molecular mechanisms involved. Based on the gene expression profile and risk grouping, the minimum gene set was determined to be 5 and the maximum gene set was determined to be 5000. Additionally, 1000 instances of re-sampling were performed. A normalized *p* value < 0.01 was considered to be statistically significant.

### 2.8. Cell Apoptosis Assay

The apoptosis of A549 cells was detected using Annexin V-FITC and a propidium iodide (PI) double staining kit purchased from Solarbio following the manufacturer’s instructions. In brief, A549 cells were seeded in a 6-well plate at 350,000 cells per well for 24 h and then treated with a vector or pcDNA3.1-CHRDL1 overexpression plasmid for another 24 h. Cells were collected and washed once with cold PBS, resuspended in 100 μL of binding buffer, and stained with Annexin V/FITC for 15 min and PI for 5 min in the dark. Then, the cells were analyzed using flow cytometry.

## 3. Results

### 3.1. Key Module Identification and Functional Enrichment Analysis

**Figure 1:**
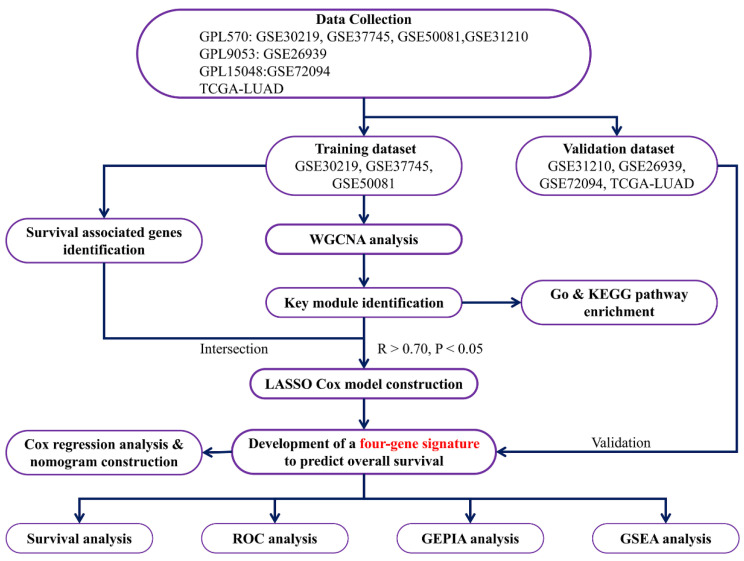
Analysis flow chart.

**Figure 2:**
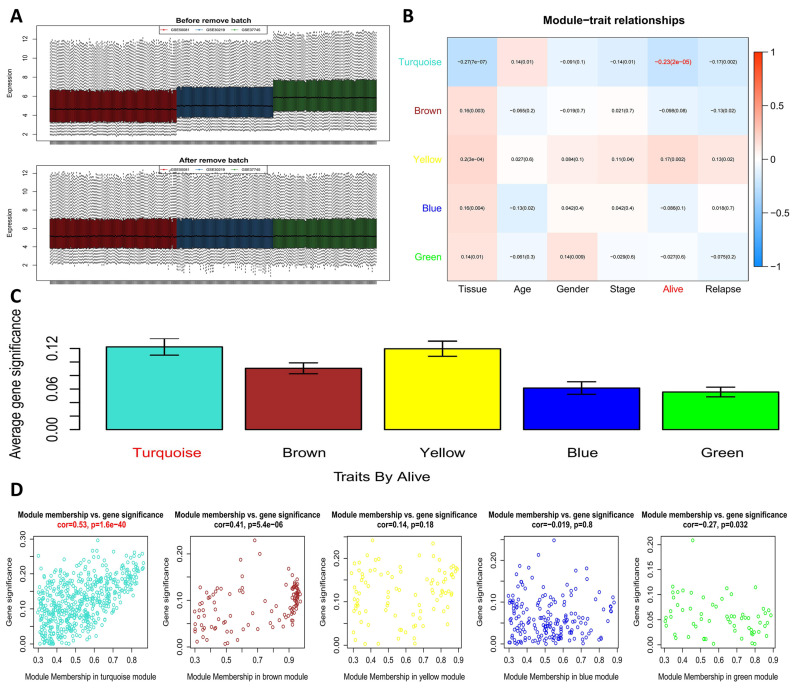
Key module identification. (**A**) Box plots of gene expression data (GSE50081, GSE30219, GSE37745) before and after normalization; (**B**) correlation between biologically significant modules and phenotypes; (**C**) average gene significance of modules; (**D**) memberships of modules.

A detailed flow chart of this study is shown in Figure 1. There were 321 samples, 54,675 genes, and 6 phenotypes in the gene expression and phenotype matrix profiles obtained from the training sets. The average RNA expression in each sample was basically the same after normalization (Figure 2A). Additionally, outlier samples were removed according to clustering distance, and then new data expression profiles, including 315 samples and 1355 genes, were acquired (Figure S1). A soft threshold of β = 4 was selected to ensure that the network was scale-free (Figure S2). After calculating the co-expression modules, genes in the new data expression profiles were allocated to five biologically significant modules. Meanwhile, the grey module represented genes that could not be aggregated into other modules (Figure S3). Furthermore, we analyzed the correlation between modules and phenotypes. It was shown that the turquoise module had the most significant correlation with “Alive” (R = −0.23, *p* = 0.00002; Figure 2B). The turquoise module was also suggested to be a key module based on the gene significance (GS) and module membership analyses (Figure 2C,D).

**Figure 3:**
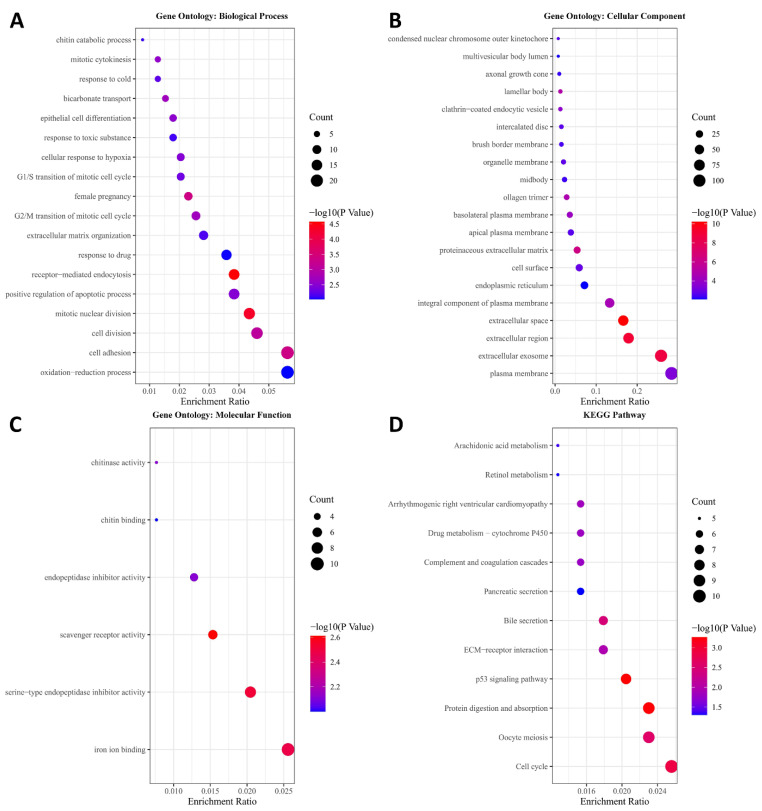
GO and KEGG pathway enrichment analysis. (**A**) Biological processes correlated with the turquoise module; (**B**) cellular components correlated with the turquoise module; (**C**) molecular functions correlated with the turquoise module; (**D**) KEGG pathways correlated with the turquoise module.

After this, GO and KEGG pathway enrichment analyses was performed for the 541 genes in the turquoise module to investigate the biological processes involved. The results indicated that various biological processes were significantly correlated with the turquoise module: mitotic nuclear division, cell adhesion, cell division, the G2/M transition of the mitotic cell cycle, mitotic cytokinesis, epithelial cell differentiation, positive regulation of the apoptotic process, and the G1/S transition of the mitotic cell cycle (Figure 3A). Additionally, cellular components correlated with the turquoise module were analyzed (Figure 3B). We found that the genes in the turquoise model might play important molecular roles in chitinase activity, chitin binding, endopeptidase inhibitor activity, scavenger receptor activity, serine-type endopeptidase inhibitor activity, and iron ion binding (Figure 3C). KEGG pathways correlated with the turquoise module, such as arachidonic acid metabolism, were investigated at this stage (Figure 3D, Table S1).

### 3.2. Modeling Gene Identification and Construction of a Four-Gene Signature for Predicting OS

**Figure 4:**
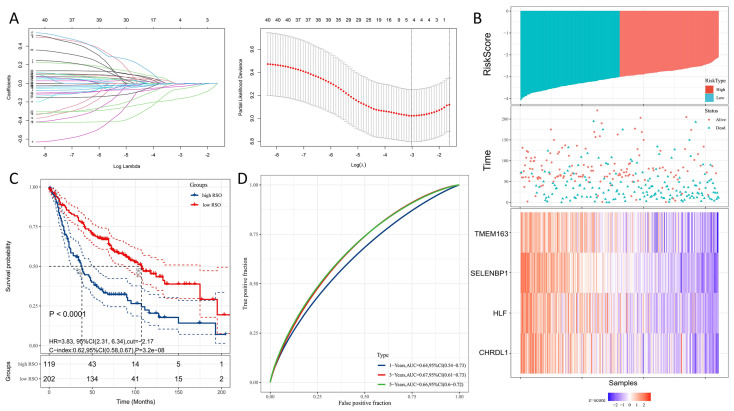
Modeling gene identification. (**A**) The relative regression coefficients of 42 genes identified by the LASSO regression analysis; (**B**) RSO scores of samples: higher levels of expression of HLF, CHRDL1, SELENBP1, and TMEM163 represented a lower risk of patient death; (**C**) univariate survival analysis of the high RSO group and low RSO group (RSO = −2.71); (**D**) ROC curves for the 1-year, 3-year, and 5-year OS.

In total, we found 12,201 genes affecting the DFS and 9488 genes associated with OS through a univariate survival analysis of the training set. We intersected these genes with the above-mentioned 541 genes identified in the turquoise module; thereby, 42 genes were acquired for further model building (Figure S4A). The relative regression coefficients of 42 genes were then calculated using LASSO regression analysis. Four genes were finally screened out for establishment of the LASSO regression model; HLF, CHRDL1, SELENBP1, and TMEM163 (Figure 4A). The detailed information of these four genes and their correlation R and *p* values are listed in Table 2. The risk score for the OS (RSO) of each sample was calculated based on the relative expression level and relative regression coefficients of these four genes.

The regression equation is as follows:

RSO = −0.03400109 × HLF expression value − 0.06167218 × CHRDL1 expression value − 0.16551196 × SELENBP1 expression value − 0.01203028 × TMEM163 expression value.

Our results suggested that these four genes are suppressor factors in LUAD (Figure 4B). A univariate survival analysis of the training sets was performed based on the RSO values (RSO = −2.71 as the cutoff); the prognosis of the higher RSO group was found to be worse than that of the lower RSO group (*p* < 0.0001, HR = 3.83, 95%CI:2.31–6.34, Figure 4C). Moreover, the ROC curve showed that the AUC values of the 4-gene signature were 0.64, 0.67, and 0.66 at 1, 3, and 5 years, respectively, indicating this 4-gene signature as a possible predictive factor of OS (Figure 4D).

### 3.3. Prognostic Value of the Four-Gene Signature

The expression profile data of four genes in the prognostic model from four validation sets (GSE26939, GSE31210, GSE72094, TCGA-LUAD) were extracted. The results shown in Table 1 indicate that the gender distribution of the GSE31210 (*p* = 0.0370) and TCGA-LUAD (*p* = 0.0066) datasets was different from that of the training dataset, and that there were more female patients. In terms of age distribution, the overall age of patients in the GSE31210 set was lower than that in the training set, while the patients in the GSE72094 set were older than those in the training set. In terms of clinical staging, the clinical staging of the four validation sets was significantly different from that of the training set. GSE26939 (*p* < 0.0001), GSE72094 (*p* < 0.0001), and TCGA-LUAD (*p* < 0.0001) all contained more advanced patients (stage III–IV). In GSE31210, there were more early stage I–II patients (*p* = 0.0050). In terms of prognosis, the GSE26939 and GSE72094 datasets did not contain the recurrence information of patients, which could not be statistically tested. However, there was no significant difference between the GSE31210 data set and the training set in terms of the number distribution of recurrent patients, while there were more recurrent patients in the TCGA-LUAD data set (*p* = 0.0360). Except for GSE26939, the number of patients who died in the other three validation sets was lower than the number who died in the training set (*p* < 0.0001).

**Figure 5:**
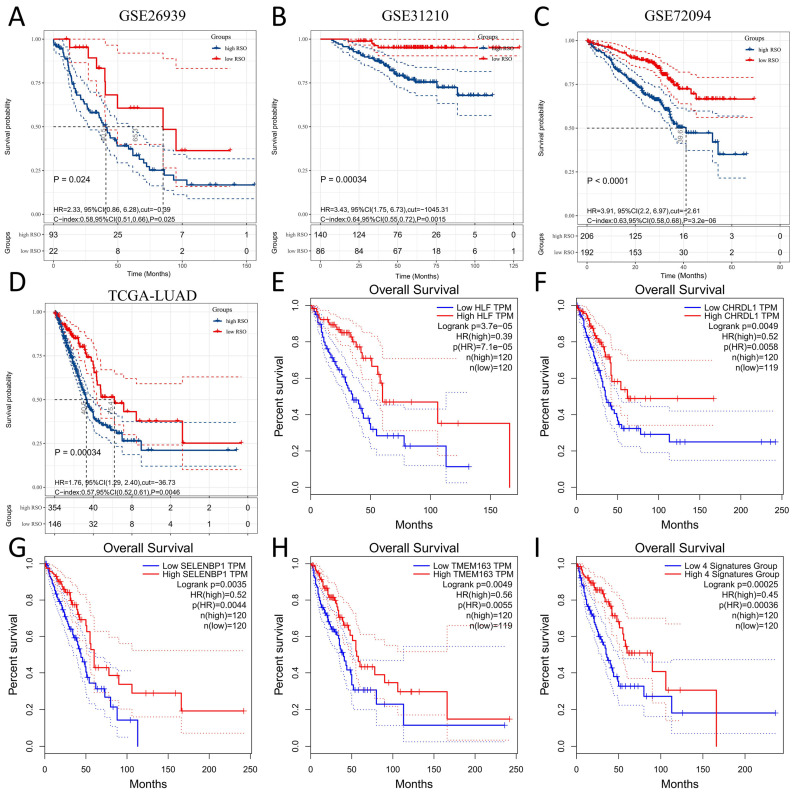
Prognostic value of the four-gene signature. (**A**) Univariate survival analysis of the high RSO group and low RSO group in GSE26939 (RSO = −0.39); (**B**) Univariate survival analysis of the high RSO group and low RSO group in GSE31210 (RSO = −1045.31); (**C**) Univariate survival analysis of the high RSO group and low RSO group in GSE72094 (RSO = −2.61); (**D**) Univariate survival analysis of the high RSO group and low RSO group in TCGA-LUAD (RSO = −36.73); (**E**) K-M survival curves of the low and high HLF groups in the GEPIA database; (**F**) K-M survival curves of low and high CHRDL1 groups in the GEPIA database; (**G**) K-M survival curves of the low and high SELENBP1 groups in the GEPIA database; (**H**) K-M survival curves of the low and high TEEM163 groups in the GEPIA database; (**I**) K-M survival curves of the low and high four-gene signature groups in the GEPIA database.

The RSO value of each sample was calculated and a univariate survival analysis was performed for each validation set. It was suggested that patients with a higher RSO had a worse prognosis, which was consistent with the training sets. Due to the different platforms and normalization methods used for each validation set, GSE26939 was cut by RSO = −0.39 (*p* = 0.024, HR = 2.33, 95%CI: 0.86–6.28, Figure 5A), GSE31210 was cut by RSO = −1045.31 (*p* = 0.00034, HR = 3.43, 95%CI: 1.75–6.73, Figure 5B), GSE72094 was cut by RSO = −2.61 (*p* < 0.0001, HR = 3.91, 95%CI: 2.2–6.97, Figure 5C), and TCGA-LUAD was cut by RSO = −36.73 (*p* = 0.00034, HR = 1.76, 95%CI: 1.29–2.40, Figure 5D). Furthermore, the AUC values of the four genes predicting the 1-year, 3-year, and 5-year OS of GSE26939 were 0.69, 0.58, and 0.53, respectively (Figure S4B), while the values for the GSE31210 were 0.64, 0.62, and 0.68 (Figure S4C). Due to the lack of follow-up time in the GSE72094 dataset, only 1-year and 3-year OS AUC values could be obtained, both of which were 0.66 (Figure S4D). The AUC values for the 1-year, 3-year, and 5-year OS for TCGA-LUAD were 0.6, 0.56, and 0.59, respectively (Figure S4E). There were one or more differences in the clinicopathological features between the four validation sets and the training set. Thus, the method used for building a prognosis model was not only available for the training set, focusing on the prediction of specific outcomes of patients with clinical pathological characteristics, but was also applicable for the other datasets, which showed obvious differences in the prognosis of patients with clinical characteristics included in predictions. To summarize, the results of this model have a certain universal application value.

The prognostic value of these four genes and integrated signatures in LUAD was further verified through the GEPIA database. We grouped the high and low expression using 75% and 25% quantile values. The HLF high expression group showed a better prognosis (*p* = 0.000037, HR = 0.39, Figure 5E). The CHRDL1 high expression group also had a better prognosis (*p* = 0.0049, HR = 0.52, Figure 5F), while the SELENBP1 and TMEM163 higher expression groups had similar results to those of another two genes (*p* = 0.0035, HR = 0.52, Figure 5G; *p* = 0.0049, HR = 0.56, Figure 5H). Significantly, the high expression group for the four-gene signature showed a better prognosis (*p* = 0.00025, HR = 0.45, Figure 5I). Furthermore, we analyzed the differences in the expression of these four genes between cancer and normal tissues via the GEPIA database. The results suggested that the expression levels of HLF, CHRDL1, and SELENBP1 in cancer tissues were significantly lower than those in normal tissues (*p* < 0.05, Figure S5). Despite there being no significant change in TMEM163 in the normal and cancer tissues, the overall expression levels of TMEM163 were higher in normal tissues than in adjacent tissues. To summarize, these four genes might act as tumor suppressor genes in lung adenocarcinoma.

### 3.4. The Four-Gene Signature Could Be a Better Prognostic Factor Than Clinical Factors in the Training Set

**Figure 6:**
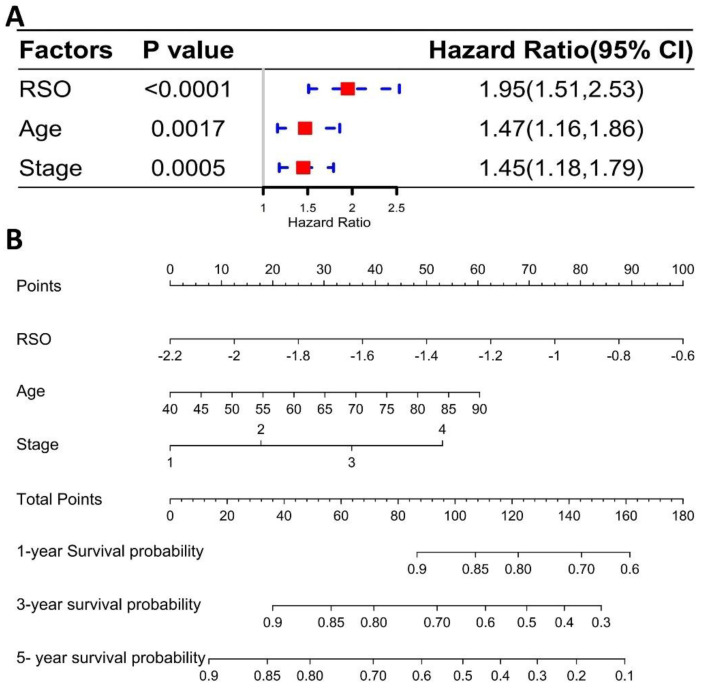
The four-gene signature is a better prognostic factor than clinical factors. (**A**) Multivariate regression analysis: the four-gene signature RSO, age, and stage; (**B**) nomograms: predicting 1-year, 3-year, and 5-year OS of LUAD patients.

In the training set, the common clinical characteristics of the three data sets were age, gender, and stage, as shown in Table 1. The RSO calculated from these four genes and the three common essential clinical factors mentioned above were included in the cox regression analysis. The univariate cox regression analysis demonstrated that RSO (*p* < 0.0001), age (*p* = 0.00428), and stage (*p* < 0.0001) were risk factors for LUAD (Figure S6A). The multivariate results showed that RSO (*p* < 0.0001), age (*p* = 0.0017), and stage (*p* = 0.0005) could be regarded as independent risk factors affecting OS (Figure 6A). RSO had a higher HR value of 1.95 (95%CI: 1.51–2.53), which implied that the risk of death in the high RSO group was 1.95 times that in the low RSO group. Additionally, the mortality of patients with a high RSO was higher than that of patients at an older age or later stage of the disease. In addition, a nomogram was built to establish a method for quantitatively predicting the probability of 1, 3, and 5-year OS in LUAD patients (Figure 6B). The RSO had a wider range of points than age or stage, indicating that RSO has a stronger ability for predicting the 1-, 3-, and 5-year survival rates. Thus, it can be concluded that the predictive value of RSO is higher than the two important clinical factors (age and stages, respectively) in the training set.

A calibration curve was then developed to analyze the optimal range of the prognostic model. This prognostic model showed a good prediction effect for 2-year OS (Figure S6B) and 3-year OS (Figure S6C). Nevertheless, the predicted OS at 2 years was lower than the actual outcome, while the predicted OS at 3 years was higher than the actual outcome. The best prediction time was at around 30 months, when the outcome of the model was closest to the actual outcome and the results had the highest degree of fit (Figure S6D).

### 3.5. Identification of Four-Gene Signature Associated Hallmark and KEGG Pathway

**Figure 7:**
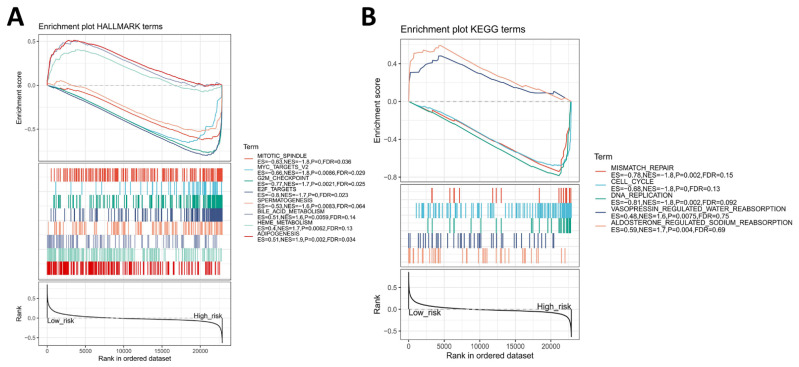
The four-gene signature-associated hallmark and KEGG pathway. (**A**) Enrichment hallmarks in the low- and high-risk groups. (**B**) Enrichment KEGG pathways in the low- and high-risk groups.

We divided the patients into a high-risk group and low-risk group in the training sets based on the cutoff for the RSO value in the training set (−2.71) and performed a GSEA (Table S2). The results suggested that the four-gene signature might be involved in the following biological processes: mitotic spindle, MYC targets, G2M checkpoint, E2F targets, bile acid metabolism, heme metabolism, and adipogenesis (Figure 7A). Moreover, the four-gene signature might regulate the following pathways: mismatch repair, cell cycle, DNA replication, vasopressin-regulated water reabsorption, and aldosterone-regulated sodium reabsorption (Figure 7B). These results were able to provide directions for further research.

### 3.6. CHRDL1 Could Accelerate the Early Apoptosis of Lung Adenocarcinoma Cell Line A549

**Figure 8:**
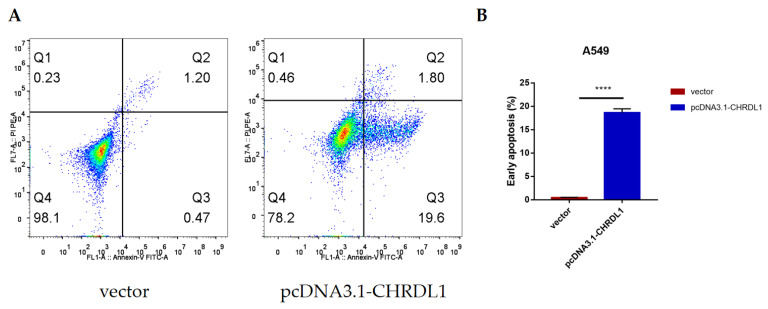
Flow cytometry analysis of A549 treated with vector and pcDNA3.1-CHRDL1. (**A**) CHRDL1 induced early apoptosis in A549 cells. (**B**) The percentage of early apoptosis. Values represent means ± SDs of three independent experiments. **** *p* < 0.0001, significantly different from the vector control.

To ascertain whether CHRDL1 was a tumor suppressor gene in lung cancer, we performed an apoptosis analysis (Figure 8). A549 cells were treated with CHRDL1 overexpression plasmids for 24 h and then examined using flow cytometry. As shown in Figure 8A, it was clear that CHRDL1 overexpression could induce the early apoptosis of A549 cells. Additionally, a significant change between the vector group and the pcDNA3.1-CHRDL1 group (*p* < 0.0001; Figure 8B) was found.

## 4. Discussion

WGCNA is a type of algorithm that is used for obtaining module information from chip data. The most variable genes in WGCNA are used for identifying interesting modules and performing significant association analyses with phenotype. WGCNA has demonstrated its superiority and specificality for screening out key modules. The most important advantage of WGCNA is its ability to perform multiple hypothesis testing elimination by converting thousands of genes and phenotypes into a smaller number of modules and phenotypes [33,34]. In our study, we established a four-gene signature for LUAD via WGCNA. We firstly integrated and analyzed three microarray datasets from GEO. A key module that significantly correlated with “Alive” was identified. The functional enrichment analysis demonstrated that the key module was enriched in various biological processes such as the cell cycle, cell differentiation, and cell apoptosis. These results were consistent with those of a previous study, which showed that the gain or loss of these functions plays important roles in LUAD tumorigenesis and progression [35].

In recent years, a number of prognostic gene signatures for lung cancer have been identified. For example, a linear prognostic model of eight genes (DLGAP5, KIF11, RAD51AP1, CCNB1, AURKA, CDC6, OIP5, and NCAPG) was built and served as a potential prognostic biomarker of LUAD [36]. Despite the fact that the authors validated the prognostic model in their hospital, AUC values under ROC curves were not presented in this study. In our study, the AUC values of GSE31210 were 0.64, 0.62, and 0.68, which were comparative with the findings of previous reports. Moreover, the gene number of this model was higher than the gene number in this study. Another nine-gene signature containing nine glycolysis-related genes (HMMR, B4GALT1, SLC16A3, ANGPTL4, EXT1, GPC1, RBCK1, SOD1, and AGRN) was established in 2019 [37]. In that particular study, only the TCGA dataset was utilized as a validation set, whereas we used four validation sets including the GSE26939, GSE31210, GSE72094, and TCGA datasets, for validating the prognostic value of the four-gene signature model in our study. A 22-gene signature and an 11-gene signature were reported to dichotomize patients with different OS significantly. These two signatures could serve as independent predictors of OS in lung adenocarcinoma and squamous cell carcinoma, respectively [38]. However, there were more genes in this report compared with in our study, so it might be more difficult for their signature to undergo clinical translation. Similar to our study, a robust six-gene signature was constructed for predicting both the DFS and OS of NSCLC patients via multivariate regression and stratification analyses [39]. In our work, we identified four hub genes associated with OS—namely, HLF, CHRDL1, SELENBP1, and TMEM163. The prognosis value of the four-gene signature was verified in four validation sets as well as in the GEPIA database. Significantly, our prognostic model for the four-gene signature can be applied in datasets with early LUAD patients (GSE31210) as well as LUAD datasets with different sequencing platforms (GSE26939, GSE72094, and TCGA-LUAD). Moreover, our four-gene signature was a better prognostic factor than clinical factors including age and stages. Furthermore, in terms of the number of genes, the number used was less than that used in the above reports, so our results may be easier to use in subsequent clinical translational research or for the development of a detection kit to promote clinical applications.

Hepatic leukemia factor (HLF) is a circadian gene that belongs to the family of the proline and acidic amino acid-rich basic leucine zipper transcription factors (PAR bZIP) [40,41]. Previous studies have demonstrated that HLF plays an essential physiological role in nervous system development [42], as well as in fibroblast apoptosis [43]. Moreover, the aberrant expression of HLF was found to extensively participate in the various processes of tumorigenesis; HLF was found to be downregulated in glioma and may promote the proliferation, metastasis, and radiosensitivity of cancer cells [44]. HLF expression was also decreased in hematological malignancy and was found to be a novel leukemic stem cell regulator [45]. On the contrary, HLF overexpression was found to promote the evolution of sorafenib resistance in patients with hepatocellular carcinomas via upregulation of OCT4 and SOX2 [46]. These findings indicated the paradoxical roles of HLF in tumors, which are tumor type-dependent. In NSCLC, HLF expression was reported to be decreased in tumor tissues. Additionally, HLF downregulation could promote multiple-organ distant metastases in NSCLC through PPAR/NF-κB signaling; thus, HLF might serve as a prognostic biomarker of NSCLC [47]. These results are consistent with those of our study and further demonstrate HLF to be a tumor suppressor factor in LUAD.

As a secreted protein, chordin-like 1 (CHRDL1) acts as an antagonist of bone morphogenetic protein (BMP)-mediated signaling via the Smad pathway [48]. Several studies have revealed that CHRDL1 plays a vital role in adult brain and embryonic cell differentiation [49,50]. CHRDL1 has also been suggested to mediate tumorigenesis; it has been found to be significantly downregulated in gastric tissues, in a methylation-mediated manner [51]. Gene silencing by methylation has been suggested to play an important role in carcinogenesis [52]. Consistently, CHRDL1 was found to be a prognostic biomarker of better outcomes in patients with breast cancer. It was found to be an inhibitor of migration and invasion induced by BMP4 [53]. However, the prognostic value of CHRDL1 in NSCLC has never been reported on before. In our study, we performed an apoptosis analysis to investigate the role of CHRDL1 in lung cancer. Apparently, CHRDL1 is a tumor suppressor gene that can induce early apoptosis in the lung cancer cell line. More experimental studies should be performed to verify the negative prognostic value of CHRDL1 in LUAD.

Selenium-binding protein 1 (SELENBP1) is highly expressed in human tissues, including lung tissue [54]. SELENBP1 expression is associated with poor prognosis in several cancer types, including lung adenocarcinoma, hepatocellular carcinoma, and colorectal carcinogenesis, as well as breast cancer [55,56,57,58]. SELENBP1 was demonstrated to be involved in the tumor growth-suppressive effects of Nkx2-1, and it was reported to inhibit tumor growth and the migration of lung adenocarcinoma [59]. A similar conclusion was obtained in our work, where SELENBP1 was found to be a suppressor factor of LUAD. These previous experimental studies further prove the reliability of our study.

Transmembrane 163 (TMEM163), also known as synaptic vesicle 31 (SV31) [60], was recently characterized as a zinc efflux transporter [61]. It was reported that TMEM163 is most highly expressed in the lungs, followed by the brain and testis [62]. There is still some controversy regarding the zinc transport function of TMEM163 (influx or efflux transporter). Initial studies conducted on this used PC12 cells for expressing rodent Tmem163 transiently or stably. These cells showed intracellular zinc accumulation when they were exposed to exogenous zinc, indicating that rodent Tmem163 is a zinc influx transporter [60]. By contrast, when transiently expressing TMEM163 in HEK-293 cells, intracellular zinc would be increased. This result implied that TMEM163 might act as an influx transporter [63]. Nevertheless, the roles of TMEM163 in tumorigenesis have never been investigated. Our study first put forward TMEM163 as a suppressor biomarker in LUAD, which requires further verification.

There are some limitations in our study. First, our study was performed only through bioinformatic analysis, meaning that further experiments are needed to validate the reliability of our results using tumor samples and clinical information. Previous experiments have already demonstrated the anti-tumor roles of HLF and SELENBP1 in LUAD [47,59]. However, the prognostic value of CHRDL1 and TMEM163 in LUAD had never before been reported. Moreover, the roles of TMEM163 in tumorigenesis have never been investigated. Thus, the molecular functions of CHRDL1 and TMEM163 in LUAD should be further investigated in in vitro and in vivo experiments. Second, it is hard to determine an accurate RSO cutoff value due to the different sequencing platforms and methods of normalization used; therefore, further large-scale prospective clinical trials need to be performed.

## 5. Conclusions

In conclusion, four genes were identified by our integrated bioinformatics analysis. Our results revealed that they were suppressor factors of LUAD and that their high expression predicted a lower risk of death. Moreover, we identified a four-gene signature as a potential prognostic factor for LUAD patients. These findings provide a theoretical basis for exploring potential biomarkers for LUAD prognosis prediction in the future.

## Figures and Tables

**Table 1:** Clinical characteristics of patients with lung adenocarcinoma in each dataset.

Characteristics	Training Dataset	Validation Dataset							
		GSE26939	*p* Value ^$^	GSE31210	*p* Value ^$^	GSE72094	*p* Value ^$^	TCGA-LUAD	*p* Value ^$^
**Number of patients**	321	116		226		442		500	
**Platforms**	GPL570	GPL9053		GPL570		GPL15048		GDC Data Portal	
**Gender**									
Male	179	49	0.2518	105	0.0370 *	240	0.7128	230	0.0066 **
Female	142	51		121		202		270	
Unknown	0	16		0		0		0	
**Age (years)**	64.90 ± 9.92	64.04 ± 10.88	0.4359	59.58 ± 7.40	<0.0001 ****	69.30 ± 9.33	<0.0001 ****	65.26 ± 10.05	0.6244
**Clinical Stage**									
Unknown	0	29		0		28		8	
Stage I	234	55	<0.0001 ****	168	0.0050 **	265	<0.0001 ****	268	<0.0001 ****
Stage II	70	14		58		69		119	
Stage III	13	16		0		63		80	
Stage IV	4	2		0		17		25	
**Relapse**									
No	174	Null		162	0.1728	Null		289	0.0360 *
Yes	91			64				211	
Unknown	56			20				0	
**Follow up**									
Alive	145	49	0.6629	191	<0.0001 ****	298	<0.0001 ****	318	<0.0001 ****
Dead	176	66		35		122		182	
Unknown	0	1		20		22		0	

^$^ Comparison with training set; * *p* < 0.05; ** *p* < 0.01; **** *p* < 0.0001.

**Table 2:** The detailed information of the four modeling genes and their correlation R and *p* values in the turquoise module.

Affy ID	Ensembl ID	Gene Symbol	Gene Description	Regression Coefficient	R	*p* Value
204753_s_at	ENSG00000108924	HLF	hepatic leukemia factor	−0.03400109	0.756241276	3.33 × 10^−62^
209763_at	ENSG00000101938	CHRDL1	chordin-like 1	−0.06167218	0.702915849	2.58 × 10^−50^
214433_s_at	ENSG00000143416	SELENBP1	selenium binding protein 1	−0.16551196	0.771245541	4.04 × 10^−66^
223503_at	ENSG00000152128	TMEM163	transmembrane protein 163	−0.01203028	0.703663266	1.84 × 10^−50^

## Data Availability

The datasets in the current study are open to the public at the NCBI Gene Expression Omnibus (GEO) and The Cancer Genome Atlas (TCGA) database.

## Supplementary Materials

The following supporting information can be downloaded at: https://www.mdpi.com/article/10.3390/genes13020238/s1, Figure S1: Sample clustering to detect outliers, Figure S2: Determination of soft-thresholding power in the weighted gene co-expression network analysis (WGCNA), Figure S3: Dendrogram of all genes clustered based on a dissimilarity measure, Figure S4. (A) Venn plots of overlapping genes in the turquoise module with genes affecting OS and DFS; (B) ROC curves of 1-year, 3-year, 5-year OS in GSE26939; (C) ROC curves of 1-year, 3-year, 5-year OS in GSE31210; (D) ROC curves of 1-year, 3-year, 5-year OS in GSE72094; (E) ROC curves of 1-year, 3-year, 5-year OS in TCGA-LUAD, Figure S5. The analysis of the differences in the expression of HLF, CHRDL1, SELENBP1 and TMEM163 between cancer and normal tissues via GEPIA database. Red indicates the expression of tumor tissue (num T = 438), gray indicates the expression of normal tissue (num N = 59), and each point represents a tissue. Log2(TPM + 1) were used for log-scale. * *p* < 0.05, Figure S6. (A) Univariate regression analysis: The four-gene signature RSO, age, stage and gender; (B) Nomogram: predicting probability 2-year OS; (C) Nomogram: predicting probability 3-year OS; (D) Nomogram: predicting probability 30-months OS. Table S1: The GO and KEGG pathways enrichment analysis base on turquoise module, Table S2: The GSEA results of hallmark and KEGG pathway enrichment analysis base on the cutoff of ROS (-2.71) in training dataset.

## References

[1] Ferlay J., Soerjomataram I., Dikshit R., Eser S., Mathers C., Rebelo M., Parkin D.M., Forman D., Bray F. (2015). Cancer incidence and mortality worldwide: Sources, methods and major patterns in GLOBOCAN 2012. Int. J. Cancer.

[2] Collisson E.A., Campbell J.D., Brooks A.N., Berger A.H., Lee W., Chmielecki J., Beer D.G., Cope L., Creighton C.J., Danilova L. (2014). Comprehensive molecular profiling of lung adenocarcinoma. Nature.

[3] Chalela R., Curull V., Enriquez C., Pijuan L., Bellosillo B., Gea J. (2017). Lung adenocarcinoma: From molecular basis to genome-guided therapy and immunotherapy. J. Thorac. Dis..

[4] Saintigny P., Burger J.A. (2012). Recent advances in non-small cell lung cancer biology and clinical management. Discov. Med..

[5] Calvayrac O., Pradines A., Pons E., Mazieres J., Guibert N. (2017). Molecular biomarkers for lung adenocarcinoma. Eur Respir. J..

[6] Yoneda K., Imanishi N., Ichiki Y., Tanaka F. (2019). Treatment of Non-small Cell Lung Cancer with EGFR-mutations. J. UOEH.

[7] Lin J.J., Shaw A.T. (2017). Recent Advances in Targeting ROS1 in Lung Cancer. J. Thorac. Oncol..

[8] Ding L., Getz G., Wheeler D.A., Mardis E.R., McLellan M.D., Cibulskis K., Sougnez C., Greulich H., Muzny D.M., Morgan M.B. (2008). Somatic mutations affect key pathways in lung adenocarcinoma. Nature.

[9] Jiang T., Fang Z., Tang S., Cheng R., Li Y., Ren S., Su C., Min W., Guo X., Zhu W. (2021). Mutational Landscape and Evolutionary Pattern of Liver and Brain Metastasis in Lung Adenocarcinoma. J. Thorac. Oncol..

[10] Dong H., Strome S.E., Salomao D.R., Tamura H., Hirano F., Flies D.B., Roche P.C., Lu J., Zhu G., Tamada K. (2002). Tumor-associated B7-H1 promotes T-cell apoptosis: A potential mechanism of immune evasion. Nat. Med..

[11] Iwai Y., Ishida M., Tanaka Y., Okazaki T., Honjo T., Minato N. (2002). Involvement of PD-L1 on tumor cells in the escape from host immune system and tumor immunotherapy by PD-L1 blockade. Proc. Natl. Acad. Sci. USA.

[12] Goodman A.M., Kato S., Bazhenova L., Patel S.P., Frampton G.M., Miller V., Stephens P.J., Daniels G.A., Kurzrock R. (2017). Tumor Mutational Burden as an Independent Predictor of Response to Immunotherapy in Diverse Cancers. Mol. Cancer Ther..

[13] Yi M., Jiao D., Xu H., Liu Q., Zhao W., Han X., Wu K. (2018). Biomarkers for predicting efficacy of PD-1/PD-L1 inhibitors. Mol. Cancer.

[14] Zhang J., Li D., Zhang Y., Ding Z., Zheng Y., Chen S., Wan Y. (2020). Integrative analysis of mRNA and miRNA expression profiles reveals seven potential diagnostic biomarkers for nonsmall cell lung cancer. Oncol. Rep..

[15] Ghafouri-Fard S., Shoorei H., Branicki W., Taheri M. (2020). Non-coding RNA profile in lung cancer. Exp. Mol. Pathol..

[16] Rousseaux S., Debernardi A., Jacquiau B., Vitte A.L., Vesin A., Nagy-Mignotte H., Moro-Sibilot D., Brichon P.Y., Lantuejoul S., Hainaut P. (2013). Ectopic activation of germline and placental genes identifies aggressive metastasis-prone lung cancers. Sci. Transl. Med..

[17] Botling J., Edlund K., Lohr M., Hellwig B., Holmberg L., Lambe M., Berglund A., Ekman S., Bergqvist M., Ponten F. (2013). Biomarker discovery in non-small cell lung cancer: Integrating gene expression profiling, meta-analysis, and tissue microarray validation. Clin. Cancer Res..

[18] Der S.D., Sykes J., Pintilie M., Zhu C.Q., Strumpf D., Liu N., Jurisica I., Shepherd F.A., Tsao M.S. (2014). Validation of a histology-independent prognostic gene signature for early-stage, non-small-cell lung cancer including stage IA patients. J. Thorac. Oncol..

[19] Leek J.T., Johnson W.E., Parker H.S., Fertig E.J., Jaffe A.E., Storey J.D., Zhang Y., Torres L.C. (2020). sva: Surrogate Variable Analysis; R Package Version 3.36.0. http://bioconductor.org/packages/3.14/bioc/html/sva.html.

[20] Wilkerson M.D., Yin X., Walter V., Zhao N., Cabanski C.R., Hayward M.C., Miller C.R., Socinski M.A., Parsons A.M., Thorne L.B. (2012). Differential pathogenesis of lung adenocarcinoma subtypes involving sequence mutations, copy number, chromosomal instability, and methylation. PLoS ONE.

[21] Okayama H., Kohno T., Ishii Y., Shimada Y., Shiraishi K., Iwakawa R., Furuta K., Tsuta K., Shibata T., Yamamoto S. (2012). Identification of genes upregulated in ALK-positive and EGFR/KRAS/ALK-negative lung adenocarcinomas. Cancer Res..

[22] Schabath M.B., Welsh E.A., Fulp W.J., Chen L., Teer J.K., Thompson Z.J., Engel B.E., Xie M., Berglund A.E., Creelan B.C. (2016). Differential association of STK11 and TP53 with KRAS mutation-associated gene expression, proliferation and immune surveillance in lung adenocarcinoma. Oncogene.

[23] Langfelder P., Horvath S. (2008). WGCNA: An R package for weighted correlation network analysis. BMC Bioinforma..

[24] Huang D.W., Sherman B.T., Lempicki R.A. (2009). Bioinformatics enrichment tools: Paths toward the comprehensive functional analysis of large gene lists. Nucleic Acids Res..

[25] Villanueva R.A.M., Chen Z.J. (2019). ggplot2: Elegant Graphics for Data Analysis, 2nd edition. Meas.-Interdiscip. Res. Perspect..

[26] Therneau T. (2021). A Package for Survival Analysis in R. R Package Version 3.2-7. https://cran.r-project.org/web/packages/survival/vignettes/survival.pdf.

[27] Friedman J., Hastie T., Tibshirani R. (2010). Regularization Paths for Generalized Linear Models via Coordinate Descent. J. Stat. Softw..

[28] Blanche P., Dartigues J.F., Jacqmin-Gadda H. (2013). Estimating and comparing time-dependent areas under receiver operating characteristic curves for censored event times with competing risks. Stat. Med..

[29] Tang Z., Li C., Kang B., Gao G., Li C., Zhang Z. (2017). GEPIA: A web server for cancer and normal gene expression profiling and interactive analyses. Nucleic Acids Res..

[30] Gordon M., Lumley T. (2020). forestplot: Advanced Forest Plot Using ‘grid’ Graphics. R Package Version 1.10. https://CRAN.R-project.org/package=forestplot.

[31] Harrell F.E. (2021). rms: Regression Modeling Strategies. R Package Version 6.2-0. https://CRAN.R-project.org/package=survival.

[32] Subramanian A., Tamayo P., Mootha V.K., Mukherjee S., Ebert B.L., Gillette M.A., Paulovich A., Pomeroy S.L., Golub T.R., Lander E.S. (2005). Gene set enrichment analysis: A knowledge-based approach for interpreting genome-wide expression profiles. Proc. Natl. Acad. Sci. USA.

[33] Carlson M.R., Zhang B., Fang Z., Mischel P.S., Horvath S., Nelson S.F. (2006). Gene connectivity, function, and sequence conservation: Predictions from modular yeast co-expression networks. BMC Genomics.

[34] Yang L., Xu Y., Yan Y., Luo P., Chen S., Zheng B., Yan W., Chen Y., Wang C. (2019). Common Nevus and Skin Cutaneous Melanoma: Prognostic Genes Identified by Gene Co-Expression Network Analysis. Genes.

[35] Liu G., Pei F., Yang F., Li L., Amin A.D., Liu S., Buchan J.R., Cho W.C. (2017). Role of Autophagy and Apoptosis in Non-Small-Cell Lung Cancer. Int. J. Mol. Sci..

[36] Li S., Xuan Y., Gao B., Sun X., Miao S., Lu T., Wang Y., Jiao W. (2018). Identification of an eight-gene prognostic signature for lung adenocarcinoma. Cancer Manag. Res..

[37] Zhang L., Zhang Z., Yu Z. (2019). Identification of a novel glycolysis-related gene signature for predicting metastasis and survival in patients with lung adenocarcinoma. J. Transl. Med..

[38] Liu Y., Wu L., Ao H., Zhao M., Leng X., Liu M., Ma J., Zhu J. (2019). Prognostic implications of autophagy-associated gene signatures in non-small cell lung cancer. Aging.

[39] Zuo S., Wei M., Zhang H., Chen A., Wu J., Wei J., Dong J. (2019). A robust six-gene prognostic signature for prediction of both disease-free and overall survival in non-small cell lung cancer. J. Transl. Med..

[40] Ferrell J.M., Chiang J.Y. (2015). Circadian rhythms in liver metabolism and disease. Acta Pharm. Sin. B.

[41] Reszka E., Zienolddiny S. (2018). Epigenetic Basis of Circadian Rhythm Disruption in Cancer. Methods Mol. Biol..

[42] Hitzler J.K., Soares H.D., Drolet D.W., Inaba T., O’Connel S., Rosenfeld M.G., Morgan J.I., Look A.T. (1999). Expression patterns of the hepatic leukemia factor gene in the nervous system of developing and adult mice. Brain Res..

[43] Suzuki K., Yoshida K., Ueha T., Kaneshiro K., Nakai A., Hashimoto N., Uchida K., Hashimoto T., Kawasaki Y., Shibanuma N. (2018). Methotrexate upregulates circadian transcriptional factors PAR bZIP to induce apoptosis on rheumatoid arthritis synovial fibroblasts. Arthritis Res. Ther..

[44] Chen S., Wang Y., Ni C., Meng G., Sheng X. (2016). HLF/miR-132/TTK axis regulates cell proliferation, metastasis and radiosensitivity of glioma cells. Biomed. Pharmacother..

[45] Wahlestedt M., Ladopoulos V., Hidalgo I., Castillo M.S., Hannah R., Sawen P., Wan H., Dudenhoffer-Pfeifer M., Magnusson M., Norddahl G.L. (2017). Critical Modulation of Hematopoietic Lineage Fate by Hepatic Leukemia Factor. Cell Rep..

[46] Musso O., Beraza N. (2019). Hepatocellular carcinomas: Evolution to sorafenib resistance through hepatic leukaemia factor. Gut.

[47] Chen J., Liu A., Lin Z., Wang B., Chai X., Chen S., Lu W., Zheng M., Cao T., Zhong M. (2020). Downregulation of the circadian rhythm regulator HLF promotes multiple-organ distant metastases in non-small cell lung cancer through PPAR/NF-κb signaling. Cancer Lett..

[48] Troilo H., Barrett A.L., Wohl A.P., Jowitt T.A., Collins R.F., Bayley C.P., Zuk A.V., Sengle G., Baldock C. (2015). The role of chordin fragments generated by partial tolloid cleavage in regulating BMP activity. Biochem. Soc Trans..

[49] Sawala A., Sutcliffe C., Ashe H.L. (2012). Multistep molecular mechanism for bone morphogenetic protein extracellular transport in the Drosophila embryo. Proc. Natl. Acad. Sci. USA.

[50] Watanabe T., Nagai A., Sheikh A.M., Mitaki S., Wakabayashi K., Kim S.U., Kobayashi S., Yamaguchi S. (2016). A human neural stem cell line provides neuroprotection and improves neurological performance by early intervention of neuroinflammatory system. Brain Res..

[51] Pei Y.F., Zhang Y.J., Lei Y., Wu W.D., Ma T.H., Liu X.Q. (2017). Hypermethylation of the CHRDL1 promoter induces proliferation and metastasis by activating Akt and Erk in gastric cancer. Oncotarget.

[52] Zhou J., Yang L., Zhong T., Mueller M., Men Y., Zhang N., Xie J., Giang K., Chung H., Sun X. (2015). H19 lncRNA alters DNA methylation genome wide by regulating S-adenosylhomocysteine hydrolase. Nat. Commun..

[53] Cyr-Depauw C., Northey J.J., Tabaries S., Annis M.G., Dong Z., Cory S., Hallett M., Rennhack J.P., Andrechek E.R., Siegel P.M. (2016). Chordin-Like 1 Suppresses Bone Morphogenetic Protein 4-Induced Breast Cancer Cell Migration and Invasion. Mol. Cell. Biol..

[54] Pohl N.M., Tong C., Fang W., Bi X., Li T., Yang W. (2009). Transcriptional regulation and biological functions of selenium-binding protein 1 in colorectal cancer in vitro and in nude mouse xenografts. PLoS ONE.

[55] Chen G., Wang H., Miller C.T., Thomas D.G., Gharib T.G., Misek D.E., Giordano T.J., Orringer M.B., Hanash S.M., Beer D.G. (2004). Reduced selenium-binding protein 1 expression is associated with poor outcome in lung adenocarcinomas. J. Pathol..

[56] Raucci R., Colonna G., Guerriero E., Capone F., Accardo M., Castello G., Costantini S. (2011). Structural and functional studies of the human selenium binding protein-1 and its involvement in hepatocellular carcinoma. Biochim. Biophys. Acta.

[57] Kim H., Kang H.J., You K.T., Kim S.H., Lee K.Y., Kim T.I., Kim C., Song S.Y., Kim H.J., Lee C. (2006). Suppression of human selenium-binding protein 1 is a late event in colorectal carcinogenesis and is associated with poor survival. Proteomics.

[58] Zhang S., Li F., Younes M., Liu H., Chen C., Yao Q. (2013). Reduced Selenium-Binding Protein 1 in Breast Cancer Correlates with Poor Survival and Resistance to the Anti-Proliferative Effects of Selenium. PLoS ONE.

[59] Caswell D.R., Chuang C.H., Ma R.K., Winters I.P., Snyder E.L., Winslow M.M. (2018). Tumor Suppressor Activity of Selenbp1, a Direct Nkx2-1 Target, in Lung Adenocarcinoma. Mol. Cancer Res..

[60] Burre J., Zimmermann H., Volknandt W. (2007). Identification and characterization of SV31, a novel synaptic vesicle membrane protein and potential transporter. J. Neurochem..

[61] Sanchez V.B., Ali S., Escobar A., Cuajungco M.P. (2019). Transmembrane 163 (TMEM163) protein effluxes zinc. Arch. Biochem. Biophys..

[62] Cuajungco M.P., Basilio L.C., Silva J., Hart T., Tringali J., Chen C.C., Biel M., Grimm C. (2014). Cellular zinc levels are modulated by TRPML1-TMEM163 interaction. Traffic.

[63] Cuajungco M.P., Kiselyov K. (2017). The mucolipin-1 (TRPML1) ion channel, transmembrane-163 (TMEM163) protein, and lysosomal zinc handling. Front. Biosci. (Landmark Ed.).

