# Supplementary material for: Identification of a Four-Gene Signature Associated with the Prognosis Prediction of Lung Adenocarcinoma Based on Integrated Bioinformatics Analysis
Source: Genes (Basel). 2022 Jan 27;13(2):238. doi: 10.3390/genes13020238 (PMC8872064; doi:10.3390/genes13020238)
Supplement: Supplementary file 1 [file genes-13-00238-s001.zip › supplemental figures.pdf]

Supplemental figures

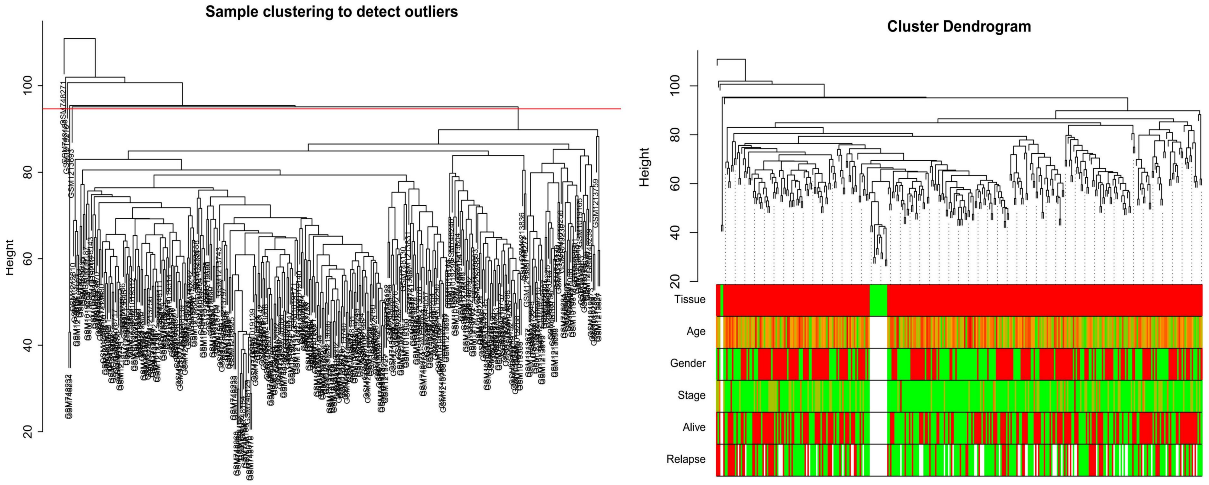

Supplementary Figure S1. Sample clustering to detect outliers

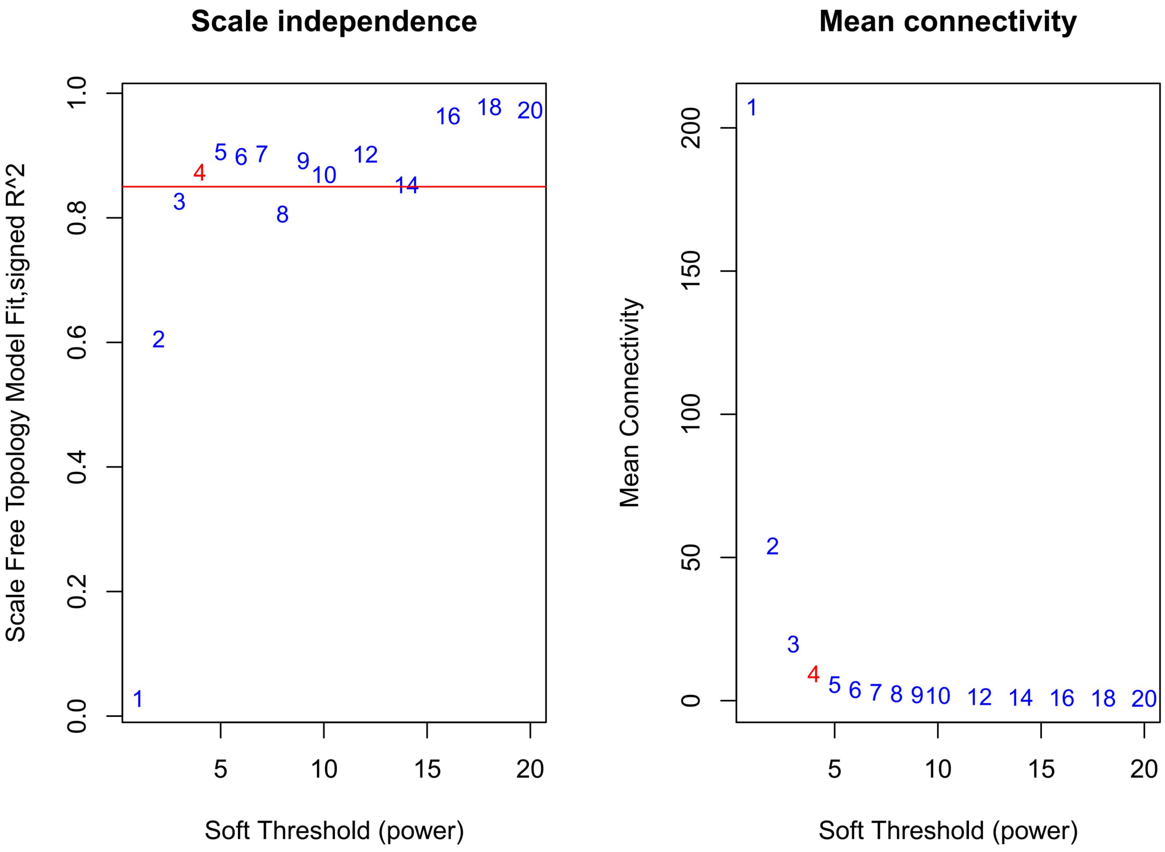

Supplementary Figure S2. Determination of soft-thresholding power in the weighted gene co-expression network analysis (WGCNA)

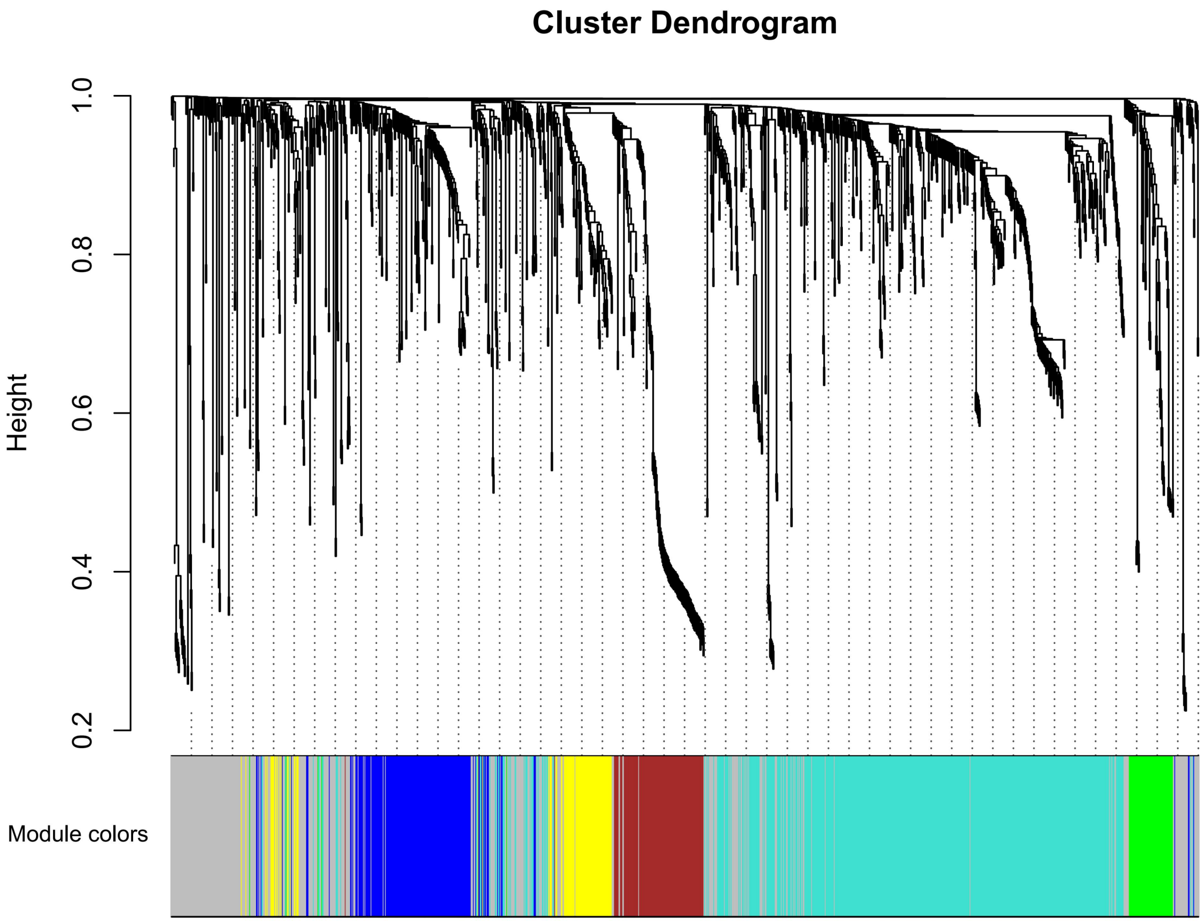

Supplementary Figure S3. Dendrogram of all genes clustered based on a dissimilarity measure

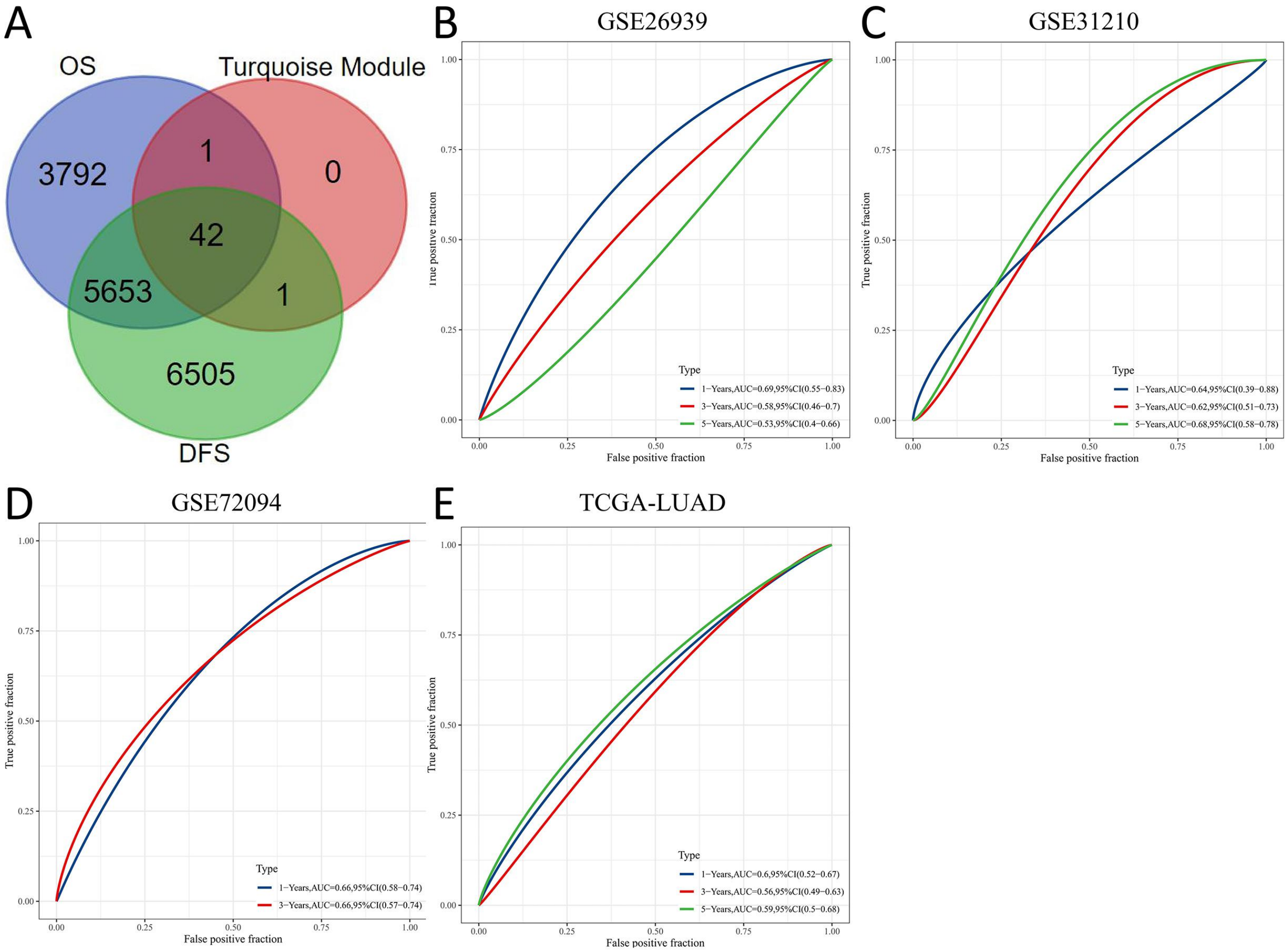

Supplementary Figure S4. (A) Venn plots of overlapping genes in the turquoise module with genes affecting OS and DFS; (B) ROC curves of 1-year, 3-year, 5-year OS in GSE26939; (C) ROC curves of 1-year, 3-year, 5-year OS in GSE31210; (D) ROC curves of 1-year, 3-year, 5-year OS in GSE72094; (E) ROC curves of 1-year, 3-year, 5-year OS in TCGA-LUAD;

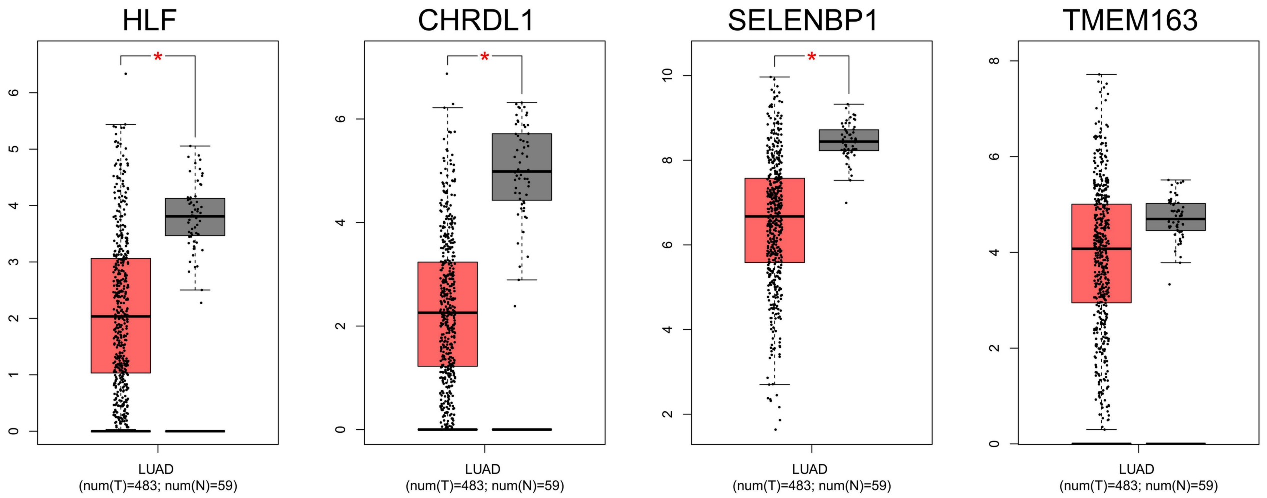

Supplementary Figure S5 The analysis of the differences in the expression of HLF, CHRDL1, SELENBP1 and TMEM163 between cancer and normal tissues via GEPIA database. Red indicates the expression of tumor tissue (num T = 438), gray indicates the expression of normal tissue (num N = 59), and each point represents a tissue. $Log_2(TPM + 1)$ were used for log-scale. * $P < 0.05$

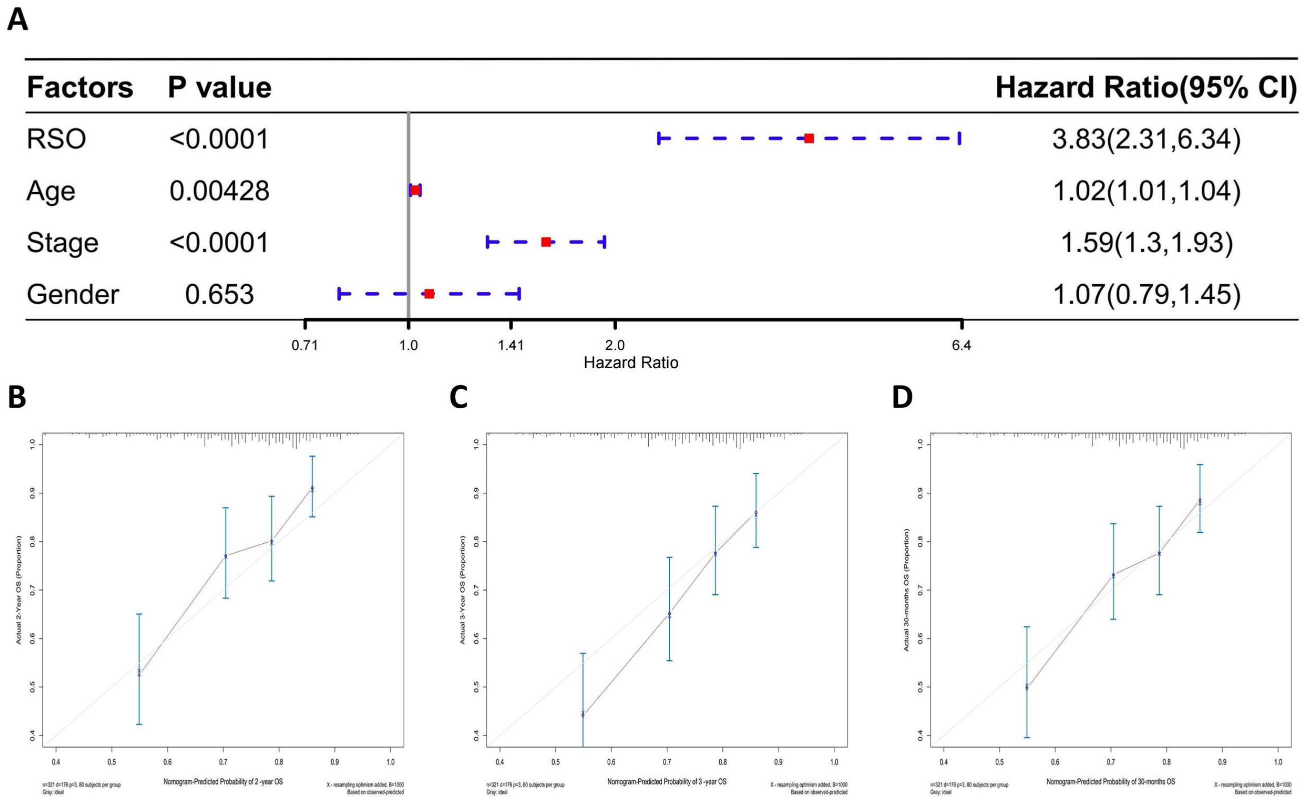

Supplementary Figure S6. (A) Univariate regression analysis: The four-gene signature RSO, age, stage and gender; (B) Nomogram: predicting probability 2-year OS; (C) Nomogram: predicting probability 3-year OS; (D) Nomogram: predicting probability 30-months OS.
