# Supplementary material for: Identification of a Four-Gene Signature Associated with the Prognosis Prediction of Lung Adenocarcinoma Based on Integrated Bioinformatics Analysis
Source: Genes (Basel). 2022 Jan 27;13(2):238. doi: 10.3390/genes13020238 (PMC8872064; doi:10.3390/genes13020238)
Supplement: Supplementary file 1 [file genes-13-00238-s001.zip › supplemental tables.pdf]

Supplemental tables

Table S1. The GO and KEGG pathways enrichment analysis base on turquoise module

Category	Term	Count	P Value	Enrichment Ratio (%)	Genes
GO_BP	chitin catabolic process	3	0.0080256	0.7672634	CHIT1, CHIA, CHI3L2
GO_BP	mitotic cytokinesis	5	0.0026289	1.2787724	ANLN, KIF4A, ANK3, CENPA, CEP55
GO_BP	response to cold	5	0.0058358	1.2787724	PLAC8, LPL, ADM, ADRB1, FOS
GO_BP	bicarbonate transport	6	0.0018775	1.5345269	CA2, HBB, SLC26A9, HBA1, SLC4A4, AQP1
GO_BP	epithelial cell differentiation	7	0.0029109	1.7902813	MUC1, EHF, DMBT1, SPINK5, CDK1, TCF21, CES1
GO_BP	response to toxic substance	7	0.0075510	1.7902813	PON3, EPHX1, PENK, CDK1, FOS, INMT, CES1
GO_BP	cellular response to hypoxia	8	0.0033517	2.0460358	CCNB1, PMAIP1, STC1, NDNF, PTN, S100B, KCNK3, AQP1
GO_BP	G1/S transition of mitotic cell cycle	8	0.0046874	2.0460358	RRM2, CCNE2, CDKN2A, CDK1, ID4, MCM10, CDC6, CDKN3
GO_BP	female pregnancy	9	0.0004420	2.3017903	CLIC5, MUC1, HPGD, SCGB1A1, FOSB, ADM, FOS, TFCP2L1, PTHLH
GO_BP	G2/M transition of mitotic cell cycle	10	0.0019069	2.5575448	CCNB2, TPX2, CCNB1, MELK, CDK1, BIRC5, NEK2, HMMR, FOXM1, AURKA
GO_BP	extracellular matrix organization	11	0.0067388	2.8132992	COL14A1, ABI3BP, COL4A4, COL4A3, SPINK5, NDNF, ICAM4, ITGB6, NPNT, CD44, SERPINB5
GO_BP	response to drug	14	0.0093022	3.5805627	CPB2, GGH, LPL, AK4, PTN, FOS, AQP1, CCNB1, GAL, SCGB1A1, CDK1, FOSB, APOD, KCNK3
GO_BP	receptor-mediated endocytosis	15	0.0000267	3.8363171	SCARA5, SUSD2, SFTPD, HBB, TMPRSS2, LRP2, HBA1, PRSS12, MARCO, DMBT1, CD207, SCGB3A2, OLR1, CD36, FOLR1
GO_BP	positive regulation of apoptotic process	15	0.0033265	3.8363171	TOP2A, CDKN2A, ZBTB16, ADM, PTN, PNMA2, S100B, CLU, MLLT11, BMP2, MELK, GAL, CNR1, PMAIP1, ECT2

GO_BP	mitotic nuclear division	17	0.0000478	4.3478261	CDCA3, CDC6, NDC80, AURKA, CDC20, ASPM, ANLN, CCNB2, TPX2, NUF2, PBK, CDK1, BIRC5, NEK2, FAM83D, BUB1, CEP55
GO_BP	cell division	18	0.0008210	4.6035806	CDCA3, UBE2C, CDCA7, CDC6, NDC80, AURKA, CDC20, CCNB2, TPX2, CCNB1, CCNE2, NUF2, CDK1, BIRC5, NEK2, FAM83D, BUB1, MAD2L1
GO_BP	cell adhesion	22	0.0004610	5.6265985	ROBO2, CX3CR1, EPHA4, SORBS2, ICAM4, SORBS1, AZGP1, PDZD2, CDH2, PCDHA1, COL4A3, HAS3, OLR1, ITGBL1, COL6A6, PGM5, ITGB6, CD36, CD44, FREM2, HABP2, EPHA3
GO_BP	oxidation-reduction process	22	0.0094370	5.6265985	EGLN3, RRM2, STEAP4, HPGD, MAOA, HHIP, TYRP1, CYP4B1, PLOD2, HSD17B6, FMO3, ALOX15B, FMO5, C15ORF48, SDR16C5, DUOX1, CH25H, BMP2, CYP4X1, CYP2B6, HSD17B2, CRYM
GO_CC	condensed nuclear chromosome outer kinetochore	3	0.0023245	0.7672634	CCNB1, BUB1, NDC80
GO_CC	multivesicular body lumen	3	0.0078182	0.7672634	SFTPB, NAPSA, SFTPC
GO_CC	axonal growth cone	4	0.0051605	1.0230179	OLFM1, GPM6A, EPHA4, FLRT3
GO_CC	lamellar body	5	0.0000103	1.2787724	SFTA3, SFTPB, SFTPC, SFTPD, SFTPA1
GO_CC	clathrin-coated endocytic vesicle	5	0.0001790	1.2787724	SFTA3, SFTPB, SFTPC, SFTPD, SFTPA1
GO_CC	intercalated disc	6	0.0019609	1.5345269	DSP, CDH2, PKP2, PGM5, ANK3, FGF13
GO_CC	brush border membrane	6	0.0034205	1.5345269	SLC34A2, TMEM27, CD36, LRP2, FOLR1, AQP1
GO_CC	organelle membrane	8	0.0017795	2.0460358	CYP2B6, CYP4X1, EPHX1, CYP4B1, FMO3, TFPI, CYP3A5, FMO5
GO_CC	midbody	9	0.0044061	2.3017903	ASPM, KIF4A, SLC2A1, CDK1, BIRC5, NEK2, ECT2, CEP55, AURKA
GO_CC	ollagen trimer	11	0.0000150	2.8132992	MARCO, FCN3, COL14A1, COL4A4, COL4A3, SFTPD, COL21A1, COL6A6, CD36, SFTPA1, GLDN
GO_CC	basolateral plasma membrane	14	0.0000694	3.5805627	DSP, HPGD, CADM1, SLC2A1, AQP4, ANK3, AQP3, SLC4A4, AQP1, CDH2, CA2, SLCO4C1, LEPR, FOLR1

GO_CC	apical plasma membrane	15	0.0022567	3.8363171	SLC34A2, SLC1A1, SLC2A1, STC1, SORBS2, LRP2, AQP1, DPP4, DUOX1, MUC1, CDH2, SCNN1B, SLC26A9, CD36, FOLR1
GO_CC	proteinaceous extracellular matrix	21	0.0000005	5.3708440	SERPINA1, COL14A1, SFTPD, MAMDC2, NDNF, NPNT, GLDN, COCH, MMP12, FLRT3, COL4A4, MGP, COL4A3, OGN, MMP28, GPC3, COL21A1, ALPL, COL6A6, SFTPA1, PI3
GO_CC	cell surface	23	0.0013885	5.8823529	ROBO2, LY6K, CPM, EPHA4, SLC46A2, SCARA5, PLA2G1B, HHIP, LPL, ANK3, PTN, HMMR, TFPI, CLU, DPP4, BMP2, APOH, KISS1R, FCER1A, SLC26A9, CD36, FOLR1, CD44
GO_CC	endoplasmic reticulum	28	0.0086796	7.1611253	SERPINA1, ATP8A1, LRRK2, PLOD2, PTN, LRP2, HSD17B6, TFPI, CLU, CLGN, TMEM100, PDZD2, PCDHA1, AGR3, APOD, MAGEA3, EPHA4, MAMDC2, SPINK5, ELOVL6, ANK3, ZDHHC11, FOS, FMO5, OLFM1, COL4A3, OGN, ERP27
GO_CC	integral component of plasma membrane	52	0.0000280	13.2992327	PIGR, STEAP4, HHIP, SLC2A1, ICAM4, AQP4, SLC4A4, AQP3, AQP1, VSIG2, FCER1A, SLC16A7, OLR1, CD36, SLC16A4, ABCC3, SLC34A2, EPHA4, SLC15A2, SCARA5, GPRC5A, CEACAM6, SCNN1B, CD44, EPHA3, CX3CR1, SLC22A3, SLC1A1, TMPRSS2, CD1E, ADRB1, AGER, CD1A, MUC1, TSPAN8, PCDHA1, FLRT3, CNR1, TSPAN7, KISS1R, GPC3, HAS3, KCNMB4, CADM1, CAV2, KCNJ15, MARCO, SLCO4C1, ATP13A4, SLC26A9, FOLR1, KCNK3
GO_CC	extracellular space	65	<0.0000001	16.6240409	PIGR, CPM, ORM1, SERPINA1, C2ORF40, COL14A1, LRRK2, CXCL17, ADM, ICAM4, C4BPA, CLU, GLDN, WFDC2, DMBT1, CA2, SOSTDC1, CD36, TPSAB1, HABP2, CPA3, SFTPB, CHIA, SFTPC, SFTPD, GKN2, PGC, SERPINB5, CHIT1, OLFM1, AZGP1, RBP4, GAL, SLPI, CEACAM6, CHI3L2, HIST1H2BG, CES1, CCL14, PON3, CPB2, PLA2G1B, LPL, STC1, PTN, TFPI, PTHLH, SELENBP1, MUC1, FLRT3, ABI3BP, APOH, GPC3, APOD, NAPSA, SERPIND1, GDF15, GGH, S100B, BMP2, SCGB1A1, OGN, SCGB3A1, ALPL, SFTPA1
GO_CC	extracellular region	70	<0.0000001	17.9028133	SFTA2, SFTA3, LY6K, ORM1, FCN3, TPSB2, SERPINA1, COL14A1, HHIP, CXCL17, HBB, ADM, ICAM4, C4BPA, CLU, CHRDL1, WFDC2, DMBT1, LEPR, TPSAB1, ENPP5, HABP2, CPA3, SFTPB, CHIA, PLA2G12B, SFTPC, IL1R2, SFTPD, SPINK5, HBA1, NPNT, CHIT1, MMP12, AZGP1, RBP4, GAL, COL4A4, COL4A3, SCG5, COL21A1, COL6A6, EPHA3, CCL14, PLA2G1B, LPL, NDNF, TFPI, AGER, MST1L, PTHLH, SCUBE2, PDZD2, PCDHA1, C7, WIF1, APOH, PENK, SUSD4, APOD, SERPIND1, GDF15, S100B, BMP2, OGN, NMU, SCGB3A2, ITGBL1, SFTPA1, FGF13

GO_CC	extracellular exosome	101	<0.0000001	25.8312021	FCGBP, STEAP4, ATP8A1, CALML3, PLOD2, CLU, SLC4A4, AQP1, DMBT1, CDH2, PEBP4, SLC15A2, SPINK5, CYS1, NPNT, PGC, AZGP1, RBP4, C1ORF116, SLPI, SCNN1B, HIST1H2BG, FREM2, GPM6A, CPB2, SLC1A1, LPL, AK4, DPP4, TMEM27, TSPAN8, C7, APOH, MYH11, APOD, S100A14, PI3, ARSE, NAPSA, CADM1, GGH, ALOX15B, OGN, CDK1, SCGB3A1, ALPL, DYNLRB2, SLC26A9, ROBO2, CPM, CLIC5, PIGR, ORM1, CLIC3, SLC44A4, SERPINA1, COL14A1, LRRK2, SLC2A1, ADIRF, HBB, WFDC2, SULT1C2, CA2, OLR1, ITGB6, CTSE, DSP, HBA1, TMC5, SERPINB5, GPRC5A, CD44, PON3, HPGD, TMPRSS2, LRP2, COCH, PBLD, SELENBP1, SCEL, MUC1, GNG4, GPC3, CRYM, SERPIND1, GDF15, SUSD2, PTPN13, PDZK1IP1, TPPP3, FABP4, MLPH, PSAT1, MGP, SLCO4C1, SCGB1A1, PI15, C16ORF89, FOLR1, CAPS
GO_CC	plasma membrane	111	0.0004210	28.3887468	LY6K, SLC46A2, STEAP4, ATP8A1, ICAM4, AQP4, C4BPA, AQP3, SLC4A4, GLDN, AQP1, CDH2, LAMP3, MAGEA1, LEPR, SLC16A7, RGS8, ENPP5, SLC34A2, EPHA4, SLC15A2, PAQR5, IL1R2, CACNA2D2, ANK3, AZGP1, MELK, CT83, SCNN1B, MALL, PKP2, FREM2, EPHA3, GPM6A, SLC22A3, PARM1, RRAD, SLC1A1, IL20RB, LPL, CACNA1D, CD1E, ADRB1, HMMR, SLC1A7, AGER, CD1A, DPP4, PCDHA1, FLRT3, PENK, KCNMB4, S100A14, STXBP6, CADM1, SYT1, CAV2, ZBTB16, ABCA8, ALOX15B, MARCO, CD207, ALPL, SLC26A9, FGF13, DIRAS3, CPM, PIGR, SLC44A4, LRRK2, FHL1, SLC2A1, TMEM100, CA2, SIX2, OLR1, FCER1A, ITGB6, CD36, ABCC3, DSP, PRSS12, GPRC5A, CEACAM6, CLDN18, HLA-DQB2, CD44, HLA-DQB1, CX3CR1, TMPRSS2, LRP2, TFPI, DUOX1, GNG4, CNR1, KISS1R, GPC3, HAS3, SCN7A, PRAME, SUSD2, KCNJ15, SORBS1, PTPN13, VEPH1, SLCO4C1, ATP13A4, ACKR1, FOLR1, CAPS, KCNK3
GO_MF	chitinase activity	3	0.0075861	0.7672634	CHIT1, CHIA, CHI3L2
GO_MF	chitin binding	3	0.0099840	0.7672634	CHIT1, CHIA, CHI3L2
GO_MF	endopeptidase inhibitor activity	5	0.0076894	1.2787724	SERPIND1, SLPI, PI3, TFPI, WFDC2
GO_MF	scavenger receptor activity	6	0.0024447	1.5345269	MARCO, SCARA5, DMBT1, SUSD2, TMPRSS2, PRSS12
GO_MF	serine-type endopeptidase inhibitor activity	8	0.0030260	2.0460358	SERPINA1, SERPIND1, SLPI, SPINK5, PI3, TFPI, SERPINB5, WFDC2
GO_MF	iron ion binding	10	0.0033134	2.5575448	CH25H, EGLN3, CYP2B6, CYP4X1, HBB, CYP4B1, PLOD2, HBA1, ALOX15B, CYP3A5

KEGG pathway	Arachidonic acid metabolism	5	0.0417296	1.2787724	HPGDS, CYP2B6, PLA2G12B, PLA2G1B, ALOX15B
KEGG pathway	Retinol metabolism	5	0.0483930	1.2787724	SDR16C5, CYP2B6, ADH1B, HSD17B6, CYP3A5
KEGG pathway	Arrhythmogenic right ventricular cardiomyopathy	6	0.0142820	1.5345269	DSP, CDH2, PKP2, CACNA2D2, CACNA1D, ITGB6
KEGG pathway	Drug metabolism - cytochrome P450	6	0.0151557	1.5345269	CYP2B6, MAOA, ADH1B, FMO3, CYP3A5, FMO5
KEGG pathway	Complement and coagulation cascades	6	0.0160644	1.5345269	CPB2, SERPINA1, C7, SERPIND1, C4BPA, TFPI
KEGG pathway	Pancreatic secretion	6	0.0495551	1.5345269	CPA3, CPB2, PLA2G12B, PLA2G1B, CA2, SLC4A4
KEGG pathway	Bile secretion	7	0.0035233	1.7902813	ABCC3, CA2, EPHX1, SLC2A1, AQP4, SLC4A4, AQP1
KEGG pathway	ECM-receptor interaction	7	0.0108365	1.7902813	COL4A4, COL4A3, COL6A6, CD36, HMMR, ITGB6, CD44
KEGG pathway	p53 signaling pathway	8	0.0005470	2.0460358	CCNB2, CCNB1, RRM2, CCNE2, CDKN2A, CDK1, PMAIP1, SERPINB5
KEGG pathway	Protein digestion and absorption	9	0.0005700	2.3017903	DPP4, CPA3, CPB2, COL14A1, COL4A4, COL4A3, SLC1A1, COL21A1, COL6A6
KEGG pathway	Oocyte meiosis	9	0.0025876	2.3017903	CDC20, CCNB2, CCNB1, CCNE2, CDK1, CALML3, BUB1, MAD2L1, AURKA
KEGG pathway	Cell cycle	10	0.0013415	2.5575448	CDC20, CCNB2, CCNB1, CCNE2, CDKN2A, CDK1, TTK, CDC6, BUB1, MAD2L1

Abbreviations: GO, Gene Ontology; BP, biological process; CC, cellular component; MF, molecular function; KEGG, Kyoto Encyclopedia of Genes and Genomes.

Table S2. The GSEA results of hallmark and KEGG pathway enrichment analysis base on the cutoff of ROS (-2.71) in training dataset

Term	ES	NES	NP	FDR	FWER
KEGG_CELL_CYCLE	-0.6837	-1.7685	<0.001	0.1324	0.23
KEGG_DNA_REPLICATION	-0.8059	-1.7643	0.002	0.0918	0.238
KEGG_MISMATCH_REPAIR	-0.7823	-1.8173	0.002	0.1509	0.161
KEGG_ALDOSTERONE_REGULATED_SODIUM_REABSORPTION	0.593	1.7217	0.004	0.6898	0.338
KEGG_VASOPRESSIN_REGULATED_WATER_REABSORPTION	0.4817	1.6453	0.0075	0.7536	0.527
HALLMARK_MITOTIC_SPINDLE	-0.6258	-1.8448	<0.001	0.0361	0.036
HALLMARK_E2F_TARGETS	-0.803	-1.722	<0.001	0.0231	0.116
HALLMARK_ADIPOGENESIS	0.5147	1.8596	0.002	0.0341	0.039
HALLMARK_G2M_CHECKPOINT	-0.7689	-1.7498	0.0021	0.0254	0.092
HALLMARK_BILE_ACID_METABOLISM	0.508	1.6138	0.0059	0.1415	0.26
HALLMARK_HEME_METABOLISM	0.4046	1.6677	0.0062	0.1328	0.184
HALLMARK_SPERMATOGENESIS	-0.5325	-1.5803	0.0083	0.0638	0.299
HALLMARK_MYC_TARGETS_V2	-0.6638	-1.7698	0.0086	0.0287	0.081

Abbreviations: GSEA, Gene Set Enrichment Analysis; ES, enrichment score; NES, normalized enrichment score; NP, normalized P value; FDR, false discovery rate; FWER, family-wise error rate; KEGG, Kyoto encyclopedia of genes and genomes.
